# Long Noncoding RNA LINC-PINT Suppresses Cell Proliferation, Invasion, and EMT by Blocking Wnt/β-Catenin Signaling in Glioblastoma

**DOI:** 10.3389/fphar.2020.586653

**Published:** 2021-01-11

**Authors:** Hanshuo Zhu, Zheng Chen, Lin Shen, Tianchi Tang, Min Yang, Xuesheng Zheng

**Affiliations:** Department of Neurosurgery, XinHua Hospital, Shanghai JiaoTong University School of Medicine, Shanghai, China

**Keywords:** glioblastoma, LINC-PINT, epithelial-mesenchymal transition, Wnt/β-catenin signaling, lncRNAs

## Abstract

**Background:** Glioblastoma (GBM) represents the most aggressive glioma with high invasive potential. Recent studies proved the involvement of epithelial-mesenchymal transition (EMT) process in increasing the malignancy and invasiveness of GBM. LncRNAs have been verified to play pivotal roles in human disease including GBM. However, the molecular mechanisms of lncRNA-mediated EMT in GBM remain largely unknown. LINC-PINT, a LncRNA which has never been studied in GBM before, was predicted to be negatively associated with EMT in GBM. This study aimed to explore the biological function and the EMT relevance of LINC-PINT in GBM and further explore the molecular mechanism.

**Methods:** The bioinformatic prediction data of LINC-PINT in GBM was derived from The Cancer Genome Atlas (TCGA) database by R software and GEPIA website. qRT-PCR assay was performed to detect the expression level of LINC-PINT in GBM cell lines. Cell counting kit-8 (CCK8), clone formation, transwell, and wound healing assays were performed to determine the biological function of LINC-PINT *in vivo*. Tumor xenograft experiment and tumor peritoneal metastasis experiments were performed to verify the *in vivo* function. Western blot and immunofluorescence staining assays were carried out to detect the relevance of LINC-PINT with EMT and Wnt/β-catenin signaling. Rescue assays were performed to check the regulation mechanism of LINC-PINT/Wnt signaling/EMT axis in GBM.

**Results:** LINC-PINT was downregulated in GBM cell lines. LINC-PINT suppressed cell progression, invasion, and EMT in GBM. LINC-PINT blocked Wnt/β-catenin signaling in GBM.

**Conclusion:** LINC-PINT suppressed cell proliferation, invasion, and EMT by blocking Wnt/β-catenin signaling in GBM.

## Introduction

Glioma is recognized to be the most common primary tumor of the central nervous system (Behin et al., 2003). WHO grade I and II glioma were clarified as low-grade glioma (LGG), while WHO grade III and IV glioma were clarified as high-grade glioma (Wesseling and Capper, 2018). The WHO grade IV glioma, characterized by its strong invasiveness, is also called glioblastoma or glioblastoma multiforme (GBM). Glioblastoma can occur primarily or develop from low-grade glioma (Ohgaki and Kleihues, 2013). In recent years, standard treatments for GBM including surgery, chemotherapy, and radiotherapy have achieved great improvement. However, total surgical resection of glioblastoma is hard to achieve due the tumors’ invasive growth into normal brain parenchyma; postoperative GBM recurrence is rather common. Actually, patients suffering from GBM are reported to have poor prognosis with an average survival time of less than 15 months and a 5-year survival rate of only 3.4% (Alexander and Cloughesy, 2017). Therefore, it is of great importance to deeply understand the molecular mechanism in GBM pathogenesis and invasiveness and to search for effective therapeutic targets.

Epithelial-mesenchymal transition (EMT), initially described in embryonic development, is a plastic, reversible, and multistep procedure that allows epithelial cells to acquire mesenchymal characteristics (Thiery et al., 2009). During EMT, tumor cells lose cell-to-cell adhesion contacts and epithelial polarity, leading to increased cell invasion and migration. EMT has been proved to be a vital cytological basis for tumor invasion and metastasis in various epithelial tumors such as breast, colorectal, pancreatic, thyroid, and lung cancer (Iwatsuki et al., 2010; Cunningham et al., 2018; Souder and Anderson, 2019). GBM tissues attribute to the mesenchymal subtype and lack of epithelial feature; E-cadherin is not expressed or very low in most GBM cells (Perego et al., 2002; Utsuki et al., 2002; Lewis-Tuffin et al., 2010; Kahlert et al., 2012). Therefore, EMT process in GBM has not been generally accepted, and the related researches are relatively low.

Long noncoding RNA is recognized as a novel class of RNA larger than 200 nucleotides that do not encode proteins. It has been verified by reliable evidence as functional biological molecule rather than “transcriptional noise” (Beermann et al., 2016). In the past few decades, LncRNAs have been proved to function in many significant cellular processes, including cell proliferation, apoptosis, metastasis, and invasion, which are closely related to the pathogenesis of various human diseases including GBM (Peng et al., 2018; Amodio et al., 2018). For example, LncRNA PAXIP1-AS1 facilitates cell invasion and angiogenesis of glioma by recruiting transcription factor ETS1 to upregulate KIF14 expression (Xu et al., 2019a). LncRNA ADAMTS9-AS2 interacts with FUS in the nucleus to inhibit MDM2-medicated FUS K48-ubiquitination and degradation, which inhibits migration and proliferation in GBM TMZ-resistant cells (Yan et al., 2019). FXR1 promotes the malignant biological behavior of glioma cells via lncRNA MIR17HG/miR-346 (miR-425–5p)/TAL1/DEC1 axis (Cao et al., 2019). Moreover, it has been verified that LncRNAs could also participate in the mediation of EMT process in GBM (Zeng et al., 2017; Zhang et al., 2017; Jia et al., 2018), but the reports are rather few and the potential biological function and molecular mechanisms of lncRNA-mediated EMT in GBM remain largely unknown.

Long intergenic non-protein coding RNA p53 induced transcript, LINC-PINT, which localizes at the human chromosome 7q32.3, has been demonstrated to act as a tumor suppressor in various kinds of cancers such as colorectal adenocarcinoma, gastric carcinoma, non-small cell lung cancer, esophageal cancer, and melanoma (Marín-Béjar et al., 2017; Feng et al., 2019; Xu et al., 2019c; Zhang et al., 2019). Moreover, LINC-PINT could also exert an oncogene role in clear cell renal cell carcinoma (Duan et al., 2019). However, the relevance and underlying mechanisms of LINC-PINT in glioblastoma have not been explored yet. In the present study, firstly, we derived the LINC-PINT data from TCGA database by R software and GEPIA website, which predicted that LINC-PINT was downregulated in GBM tissues and associated with good prognosis in GBM patients. Further gene ontology (Go) analysis and gene relevance analysis predicted that LINC-PINT could activate cell adhesion molecular binding and had negative correlation with EMT related genes N-cadherin, Vimentin, and Slug in GBM. Then we detected the expression level of LINC-PINT in human GBM cell lines and further explored the biological functions, the EMT relevance, and the molecular mechanisms of LINC-PINT in GBM. We firstly demonstrated that LINC-PINT was downregulated in GBM cell lines and could suppress tumor proliferation, invasion, and epithelial-to-mesenchymal transition by blocking Wnt/β-catenin signaling in GBM. Our findings showed a novel regulation mechanism of LINC-PINT/Wnt signaling/EMT axis in GBM, providing new perspectives into the pathogenesis and invasiveness of glioblastoma and verifying LINC-PINT as a potential prognostic biomarker and novel therapeutic target in GBM.

## Materials and Methods

### Bioinformatic Analysis

The data of LINC-PINT expression level, Kaplan–Meier survival curves, and gene correlation analysis were derived from GEPIA website (http://gepia.cancer-pku.cn), a web server for cancer and normal gene expression profiling and interactive analyses developed by Tang et al. (2017). The relevance of LINC-PINT expression with the pathological stage and recurrence and the GO analysis were analyzed using R software (ver. R 3.5.0; R Development Core Team, Vienna, Austria) from TCGA database (https://tcga-data.nci.nih.gov/tcga).

### Cell Culture

The U87, LN229, U373, A172, U251, T98, and U118 GBM cell lines and normal human astrocytes SVG p12 were purchased from Cell Bank of the Chinese Academy of Sciences in Shanghai, China. Cells were cultivated in DMEM (LN229, A172, U251, T98, and U118), MEM (U87), or 1640 (U373) (Thermo Fisher/Gibco, Carlsbad, United States), supplemented with 10% fetal bovine serum (FBS; Thermo Fisher/Gibco, Carlsbad, United States). Cells were cultured at 37°C with 5% CO_2_ and 100% humidity.

### Cell Constructions and Regent

For upregulation of LINC-PINT, the cDNA (BC130416) of LINC-PINT was established into the pcDNA3.1 vector and then transfected with NanoEnter transfection regent (NCM biotech, Suzhou, China). For stable upregulation, the lentiviral package of LINC-PINT was purchased from Vigene Biosciences (Shandong, China). For downregulation of LINC-PINT, cells were transfected with silencer for LINC-PINT (si-PINT) which was composed of a small interfering RNA (siRNA) (Ribo, Shenzhen, China) and an antisense oligonucleotide (ASO) (Ribo, Shenzhen, China) using Rfect transfection reagent (BaiDai, Changzhou, China) (Zou et al., 2018; Xu et al., 2019b). For Wnt/β-catenin pathway activation, cells under different transfection for 24 h were then treated with 20 mmol/L Lithium chloride (LiCL, Cat. #213233, Sigma, United States) for 24 h. Below are the sequences of silencer for LINC-PINT (si-PINT).

### RNA Extraction, Reverse Transcription, and qRT-PCR Assay

Total RNA of the cells was extracted by TRIzol (TaKaRa, Dalian, China) reagent. Then a total of 1,000 ng RNA was reverse-transcribed into cDNA in a volume of 20 µL with PrimeScript RT reagent kit (Takara, China). The real-time quantitative PCR was performed to test the relative mRNA expression at Applied Biosystems VIIA7 (Thermofisher, United States). The expression levels of LINC-PINT relative to Actin were calculated using the 2-ΔΔCt method as previously reported (Matyi et al., 2018; Braun et al., 2019; Kiss et al., 2019). The primer sequences were listed as follows, taking Actin as an internal control.

### CCK8 and Clone Formation Assay

In CCK8 assay, after 48 h of transfection, U87 and LN229 cells were digested and then seeded into 96-well culture plates in 100 μL medium at a density of 1 × 10^3^ cells/well. 10 μL CCK8 regent (Beyotime, Shanghai, China) was added to each well for 1 h incubation according to the manufacturer’s instructions. The optical density (OD) was measured at 450 nm with a microplate reader (BioTek, Winooski, VT, United States). In clone formation assay, after 48 h of transfection, LN229 cells were digested and seeded into 6-well plates at a density of 500 cells per well. The cells were cultivated in DMEM for two weeks with the medium being renovated every 3 days. Then the colonies were fixed in 4% paraformaldehyde for 30 min and stained with 0.1% crystal violet for 5 min. The number of colonies was calculated by photoshop software.

### Transwell Invasion and Wound Healing Assay

24-well transwell invasion plates (Corning, United States) precoated with Matrigel (BD, United States) were used for transwell invasion assay. Cell suspensions containing 2 × 10^5^ cells were transferred to the upper chamber in 0.1 ml serum-free DMEM or MEM after 48 h of transfection. 500 μL DMEM or MEM supplemented with 10% FBS was transferred to the lower chamber as a chemoattractant. The staining process was performed after 24 h (LN229) or 48 h (U87) of incubation. We fixed the cells on the lower surface of the filters in 4% paraformaldehyde for 20 min and then stained the cells with crystal violet for 20 min. Cells from five randomly picked fields were visualized. In wound healing assay, after 48 h of transfection, cell culture was continued until the cells reached a confluence of at least 90%. Then the wound healing assay was performed by scratching cell monolayers with a 200 µL plastic pipette tip along a ruler in a straight direction vertical to the transverse line. Photographs were taken (Leica DMIL LED inverted microscope) to calculate the mean number of migrating cells at 0 and 24 h. All the assays were repeated for three times.

### Western Blot Assay

The proteins were extracted by lysing the cells with RIPA lysis buffer (Solarbio, China) and protease inhibitors (Roche Applied Science, Switzerland). Then we quantified the total protein extracts using the bicinchoninic acid protein assay kit (Beyotime, Shanghai, China). Next, we separated the protein lysates with 6% or 12% SDS-PAGE and then transferred the separated proteins to 0.45 mm NC membranes. After being blocked with 5% nonfat milk at room temperature for 2 h and incubated with primary antibodies at 4°C overnight, the membranes were incubated with horseradish peroxidase-labeled secondary antibody (1:1,000, Beyotime, Shanghai, China). The primary antibodies used were anti-N-cadherin, anti-Vimentin, anti-Slug, anti-β-catenin, anti-C-Jun, anti-TCF1/TCF7, anti-CD44 (1:1,000, Cell Signaling Technology, USA), and anti-GAPDH (1:1,000, Beyotime, Shanghai, China).

### Immunofluorescence Staining

LN229 cells under different transfection were cultivated on glass coverslips in six-well plates for 72 h and were then fixed in 4% paraformaldehyde for 30 min. The fixed cells were permeabilized with 0.1% Triton X-100 in PBS for 10 min and blocked in 2% bovine serum albumin (BSA) for 1 h. After being incubated at 4°C with primary antibodies Vimentin (1:100, Cell Signaling Technology, United States) and β-catenin (1:100, Cell Signaling Technology, United States) overnight, the coverslips were washed 3 times and incubated in secondary antibody (Abcam, United States) at room temperature for 1 h. The fluorescence microscopy (Olympus BX51) was used to observe the immunofluorescence staining result.

### Tumor Xenograft and Tumor Peritoneal Metastasis Experiment

Mice were randomly divided into two groups, three for negative control group (transfected with empty vector) and three for LINC-PINT upregulation group (transfected with LINC-PINT lentiviral package). For tumor xenograft experiment, each 4-week-old male nude mouse was subcutaneously injected with transfected U87 cells (100 μL, 2 × 10^6^). After four weeks, mice were sacrificed and tumors were excised and weighed. For tumor peritoneal metastasis experiment, we injected transfected U87 cells (100 μL, 2 × 10^6^) into each four-week-old male nude mouse intraperitoneally. After four weeks, mice were sacrificed to calculate peritoneal metastatic nodules. All animal experiments were performed in animal laboratory center of Xinhua Hospital (Shanghai JiaoTong University School of Medicine, Shanghai, China). The study protocol was approved by the Animal Care and Use committee of Xinhua Hospital.

### Statistical Analysis

The statistical analysis was calculated by SPSS20.0 (Chicago, United States). The data were expressed as mean ± standard deviation. The differences between each group were calculated using Student’s t-test. *p* < 0.05 was received to be statistically significant and all the *p*-values were two-sided.

## Results

### LINC-PINT Was Downregulated in Glioblastoma Tissues and Acted as a Tumor Suppressor by Bioinformatic Prediction

**FIGURE 1:**
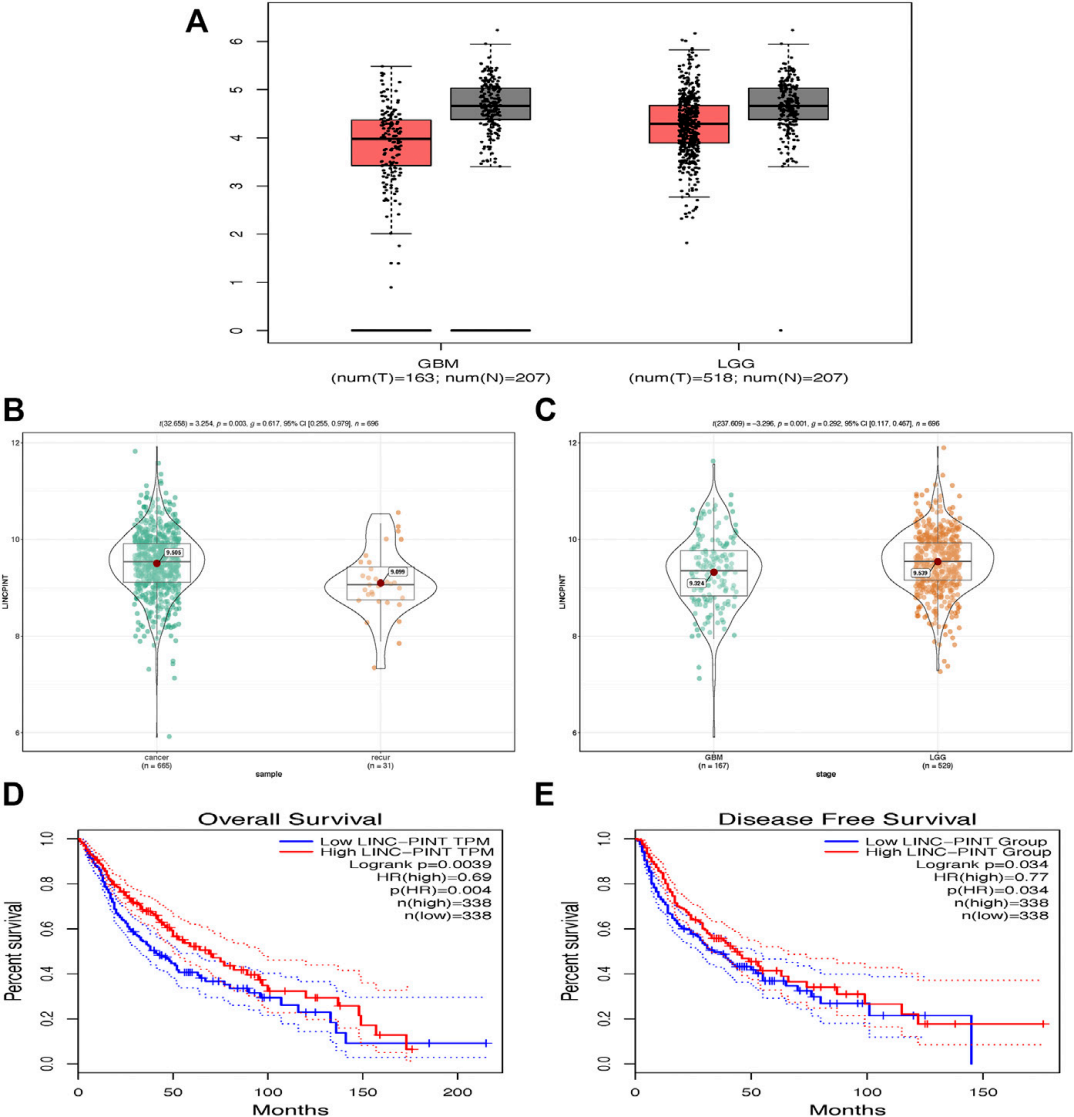
LINC-PINT was predicted to be downregulated in GBM tissues and associated with good prognosis by GEPIA database. **(A)** LINC-PINT was downregulated in LGG and GBM tissues compared to normal brain tissues. **(B)** The expression level of LINC-PINT was higher in primary glioblastoma than in recurrent glioblastoma. **(C)** The expression level of LINC-PINT was higher in LGG tissues than in GBM tissues. **(D)** Overall survival curve for GBM patients with high or low expression level of LINC-PINT. **(E)** Disease-free survival curve for GBM patients with high or low expression level of LINC-PINT.

In the present study, firstly, we derived the LINC-PINT data from TCGA database by R software and GEPIA website. It was predicted that LINC-PINT was downregulated in GBM and LGG tissues compared to normal brain tissues (Figure 1A). Moreover, primary glioblastoma showed higher expression level of LINC-PINT than recurrent group (Figure 1B) and LGG showed higher expression level of LINC-PINT than GBM group (Figure 1C), indicating that LINC-PINT might act as a tumor suppressor in GBM. In addition, Kaplan–Meier curves for overall survival (Figure 1E) and disease-free survival (Figure 1F) showed that patients with high expression level of LINC-PINT lived a good survival time, which further predicted that LINC-PINT might be a tumor suppressor in GBM.

### LINC-PINT Was Downregulated in Glioblastoma Cell Lines and Suppressed Tumor Proliferation and Viability in Glioblastoma *in vitro*

**FIGURE 2:**
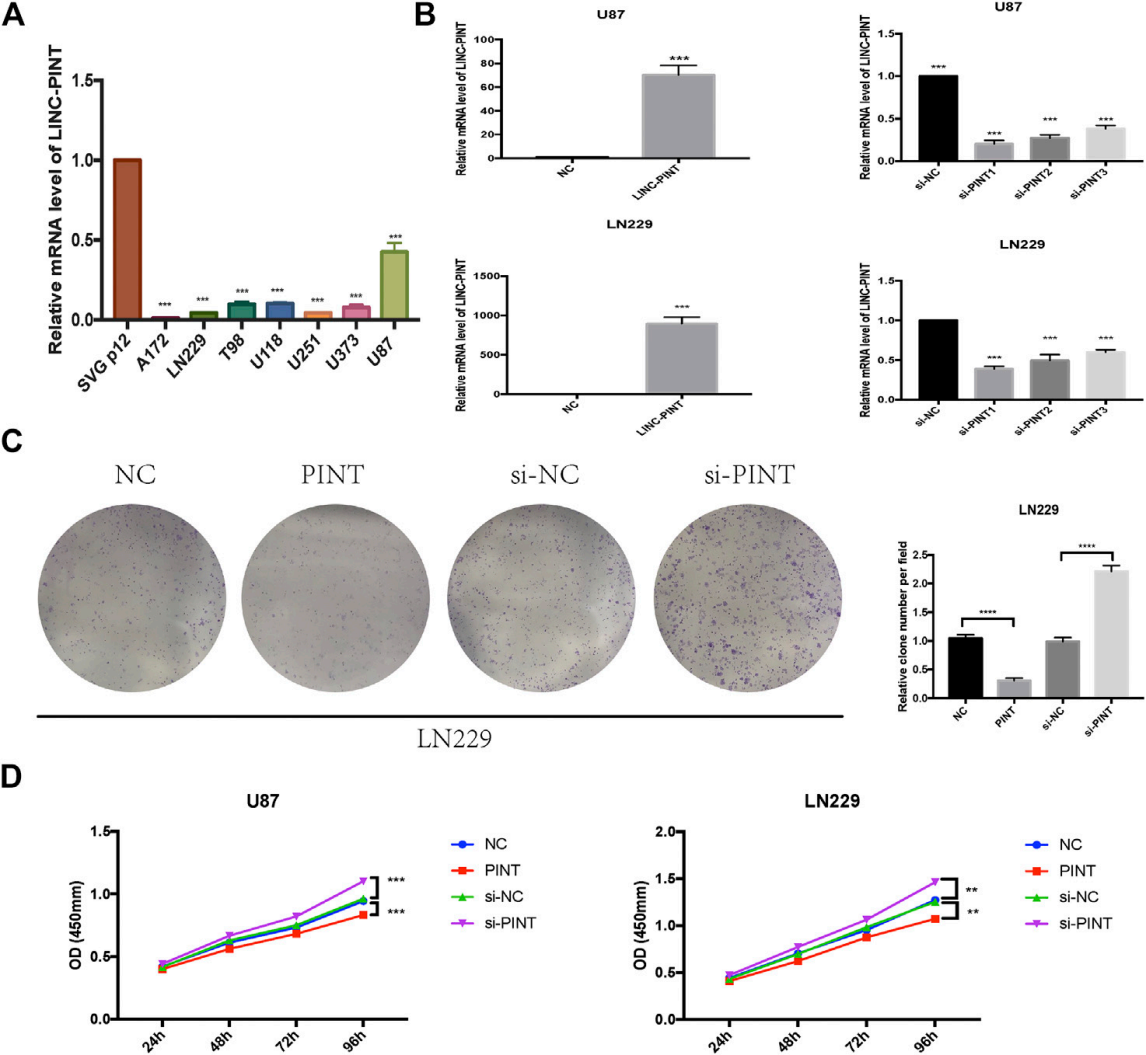
LINC-PINT was downregulated in GBM cell lines and suppressed tumor proliferation in GBM *in vitro*. **(A)** The real-time quantitative PCR assay was performed to detect the expression level of LINC-PINT in GBM cell lines A172, LN229, T98, U118, U251, U373, and U87 and normal human astrocytes SVG p12. **(B)** Upregulation and knockdown of LINC-PINT in U87 and LN229 cell lines. **(C)** Colony formation assay was performed to determine the cloning ability of LN229 cells under different transfection. **(D)** The cell viability of U87 and LN229 cells under different transfection was detected by CCK8 assay. **p < 0.01, ***p < 0.001, ****p < 0.0001.

Experimentally, we performed qRT-PCR assay to test the expression level of LINC-PINT in GBM cell lines LN229, U87, A172, T98, U373, and U251 U118 and normal human astrocytes SVG p12. And it turned out that the expression of LINC-PINT was lower in GBM cell lines than in normal human astrocytes (Figure 2A), consistent with the GEPIA database. Then we carried out gain and loss assay to testify the biological function of LINC-PINT in U87 and LN229 GBM cell lines (Figure 2B). Si-PINT1 has the largest knockdown efficiency and was used in the following experiments. Then we performed colony formation assay (Figure 2C) in LN229 cells and CCK8 assay (Figure 2D) in U87 and LN229 cell lines to determine the effect of LINC-PINT on tumor cell proliferation and viability in GBM. And it turned out that LINC-PINT inhibited cell proliferation and viability of GBM cell lines *in vitro*.

### LINC-PINT Suppressed Tumor Invasion and Migration of Glioblastoma Cells *in vitro*

**FIGURE 3:**
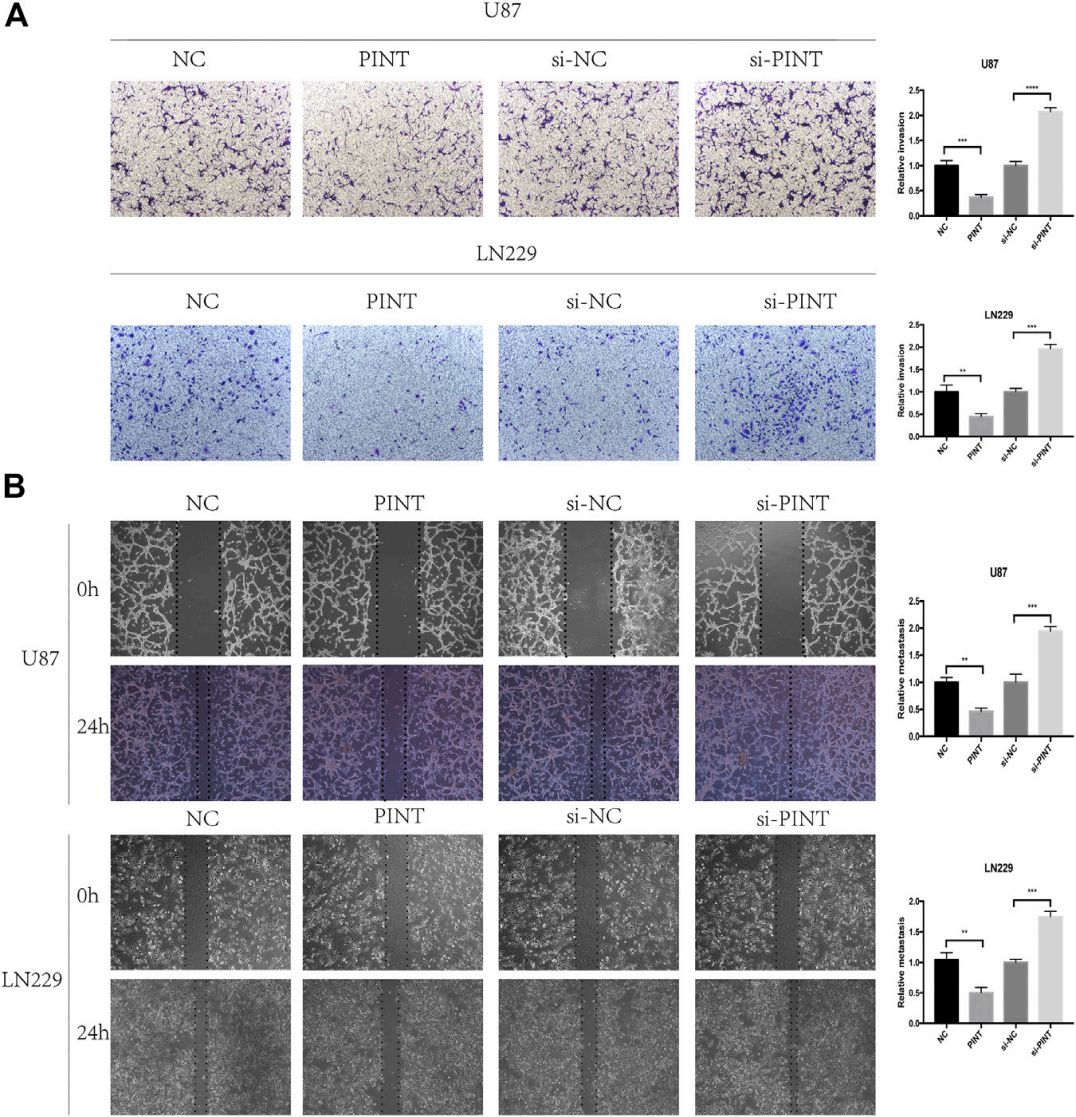
LINC-PINT suppressed cell invasion and migration of GBM cell lines *in vivo*. **(A)** The cell invasion ability of U87 and LN229 cells under different transfection was detected by transwell assay. **(B)** The wound healing assay was performed in transfected U87 and LN229 cells to determine the cell migration ability. **p < 0.01, ***p < 0.001, ****p < 0.0001.

GBM represents the most aggressive glioma with high invasive potential, infiltrating deeply into the surrounding brain parenchyma, which makes it hard to achieve total surgical resection of the tumor and postoperative glioblastoma recurrence is rather common (Alexander and Cloughesy, 2017). It is of great importance to discover new perspectives into the invasiveness of glioblastoma. So next, we carried out cell invasion assay (Figure 3A) and wound healing assay (Figure 3B) to detect the effect of LINC-PINT on GBM cell invasion and migration. The results showed that overexpression of LINC-PINT suppressed tumor invasion and migration and downregulation of LINC-PINT increased tumor invasion and migration in U87 and LN229 GBM cell lines. In summary, LINC-PINT suppressed tumor invasion and migration of GBM cell lines *in vitro*.

### LINC-PINT Suppressed Tumor Invasion and Migration of Glioblastoma Cells *in vivo*

**FIGURE 4:**
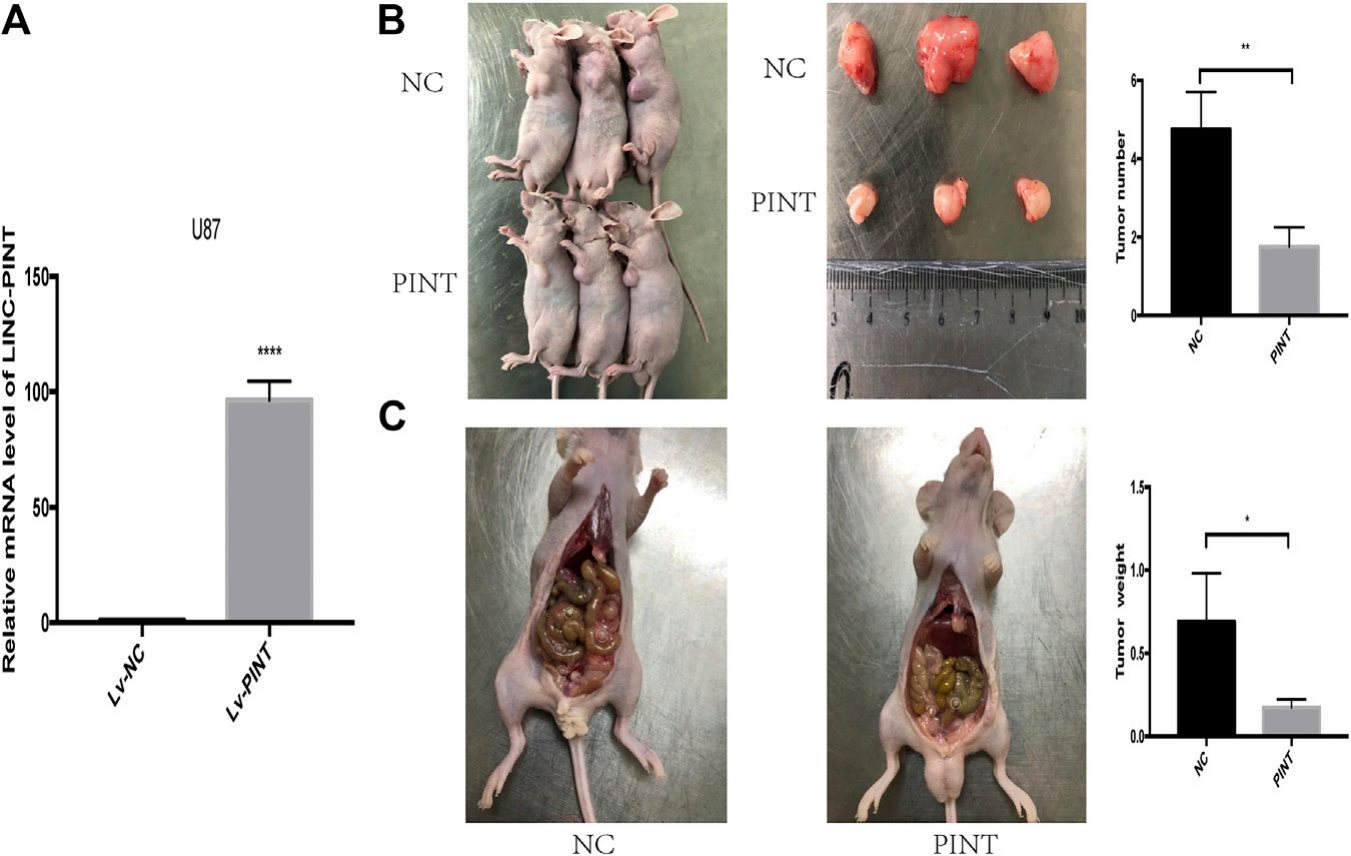
LINC-PINT suppressed tumor invasion and migration of GBM cell lines *in vivo*. **(A)** Stable upregulation of LINC-PINT in U87 cells by lentiviral package. **(B)** Tumor xenograft experiment was performed to testify GBM cell proliferation *in vivo*. **(C)** Tumor peritoneal metastasis experiment was performed to testify GBM cell invasion *in vivo.* **p < 0.01, ***p < 0.001, ****p < 0.0001.

The previous experiment showed that LINC-PINT suppressed tumor invasion and migration of GBM cell lines *in vitro*, and then we performed further tumor xenograft experiment and tumor peritoneal metastasis experiment to testify the biological function of LINC-PINT in GBM *in vivo*. We chose the U87 cells which are of good tumorigenicity and upregulated LINC-PINT in U87 cells with lentivirus (Figure 4A). As shown in tumor xenograft experiment, LINC-PINT suppressed GBM proliferation *in vivo* (Figure 4B). What’s more, tumor peritoneal metastasis experiment proved that LINC-PINT suppressed GBM invasion *in vivo* (Figure 4C).

### LINC-PINT Inhibited Epithelial-Mesenchymal Transition in Glioblastoma

**FIGURE 5:**
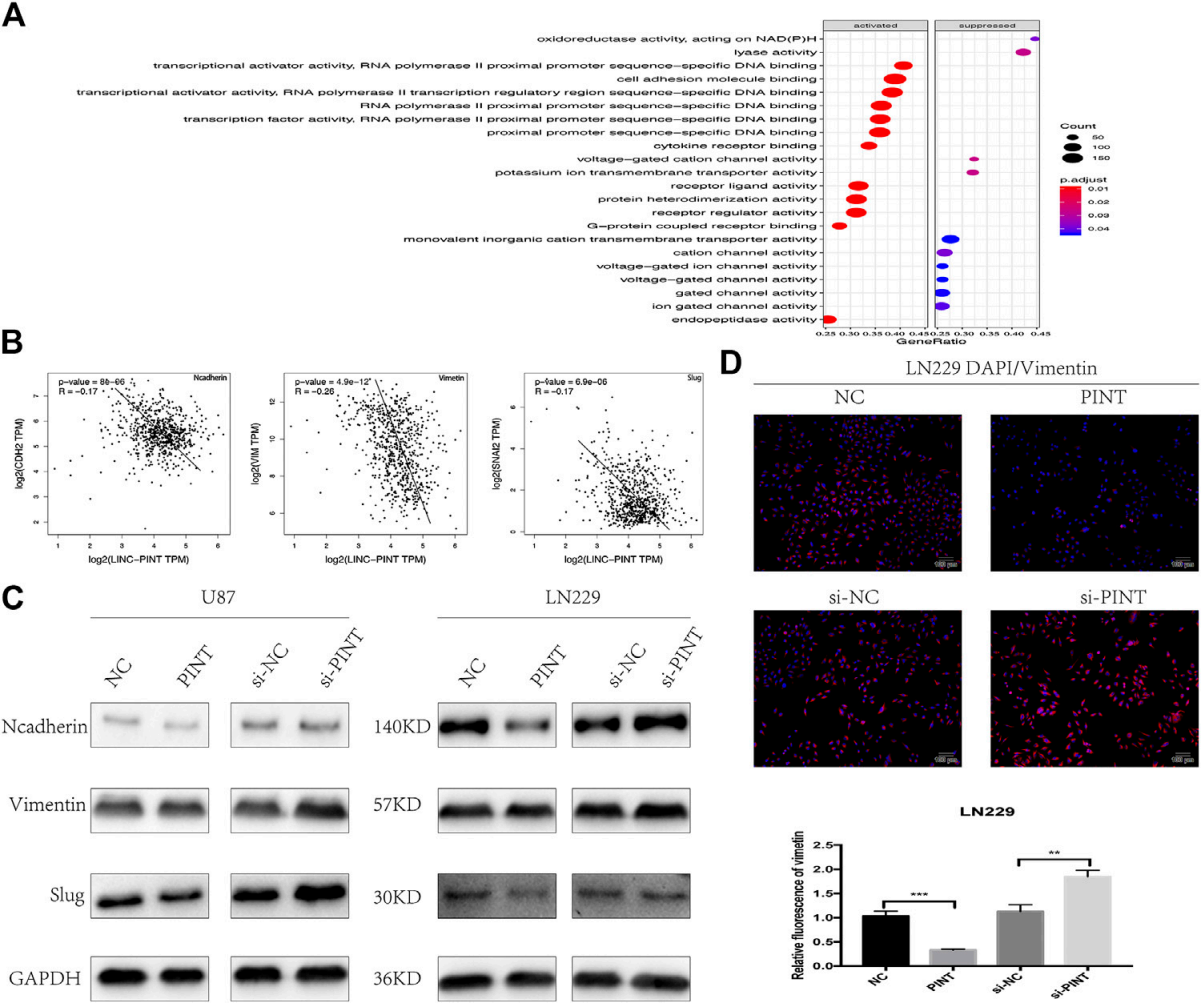
LINC-PINT inhibited epithelial-to-mesenchymal transition in GBM cell lines. **(A)** GO analysis for the potential biological functions of LINC-PINT in GBM. **(B)** The gene correlation analysis from GEPIA database predicted negative correlation of LINC-PINT with EMT markers N-cadherin, Vimentin, and Slug in GBM. **(B)** Western blot assay for EMT related markers N-cadherin, Vimentin, and Slug detected in U87 and LN229 cells under different transfection. **(C)** Representative Vimentin (red) staining in transfected LN229 cells was observed by the fluorescence microscope. Nuclei are counterstained by DAPI (blue). **p < 0.01, ***p < 0.001.

The GO analysis indicated that one of the vital biological functions LINC-PINT has was to activate the cell adhesion molecule binding (Figure 5A). As is widely recognized, losing cell adhesion contact is the main feature of epithelial-mesenchymal transition, which implied that there might be connections between LINC-PINT and EMT. What’s more, the gene correlation analysis from GEPIA database revealed negative correlation of LINC-PINT with EMT related markers N-cadherin, Vimentin, and Slug (Figure 5B). So we performed western blot assay to determine the relevance of LINC-PINT and EMT in U87 and LN229 GBM cell lines. It turned out that the expression of relevant EMT markers N-cadherin, Vimentin, and Slug was decreased by overexpression of LINC-PINT and was increased by downregulation of LINC-PINT in U87 and LN229 GBM cell lines (Figure 5C). In addition, the immunofluorescence staining of Vimentin in LN229 cells showed a parallel result with this (Figure 5D). Taken together, LINC-PINT inhibited cell epithelial-mesenchymal transition in GBM.

### LINC-PINT Blocked Wnt/β-Catenin Signaling in Glioblastoma

**FIGURE 6:**
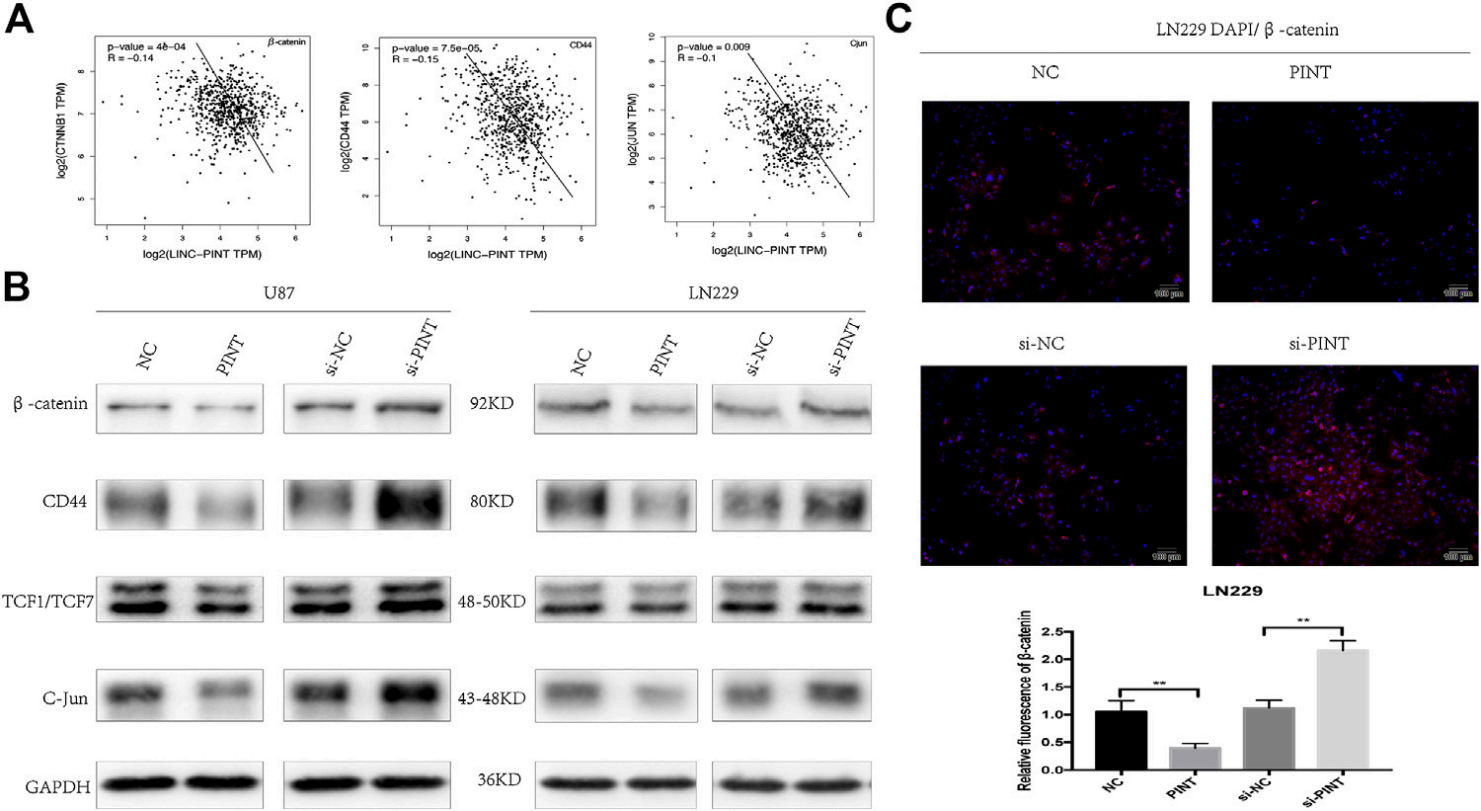
LINC-PINT blocked Wnt/β-catenin signaling pathway in GBM cell lines. **(A)** The gene correlation analysis from GEPIA database predicted negative correlation of LINC-PINT with Wnt/β-catenin related genes β-catenin, CD44, and C-Jun. **(B)** Western blot assay for Wnt/β-catenin related proteins β-catenin, CD44, TCF1/TCF7, and C-Jun detected in U87 and LN229 cells under different transfection. **(C)** Representative β-catenin (red) staining in transfected LN229 cells was observed by the fluorescence microscope. Nuclei are counterstained by DAPI (blue). **p < 0.01.

Wnt/β-catenin signaling has been testified as a classical pathway that promotes tumor progression, invasion, and migration in GBM (Zhang et al., 2012). The gene correlation analysis predicted that LINC-PINT had negative correlation with the genes β-catenin, CD44, and C-Jun related to Wnt/β-catenin pathway (Figure 6A). So we speculated that there might be some relationship between LINC-PINT and Wnt/β-catenin signaling. Then we performed western blot assay in U87 and LN229 GBM cell lines to check the effect. As demonstrated in the picture, upregulation of LINC-PINT suppressed the expression level of the relevant Wnt/β-catenin markers including the key effector β-catenin, the nuclear transcription factor TCF1/TCF7, and the downstream targets C-Jun and CD44. At the same time, silencing of LINC-PINT increased the expression of these proteins (Figure 6B). Meanwhile, the immunofluorescence staining of β-catenin in LN229 cells acquired a similar result (Figure 6C). In summary, LINC-PINT inhibited Wnt/β-catenin signaling pathway in GBM cell lines.

### LINC-PINT Suppressed Epithelial-Mesenchymal Transition, Tumor Proliferation, and Invasion Through Wnt/β-Catenin Signaling in Glioblastoma

**FIGURE 7:**
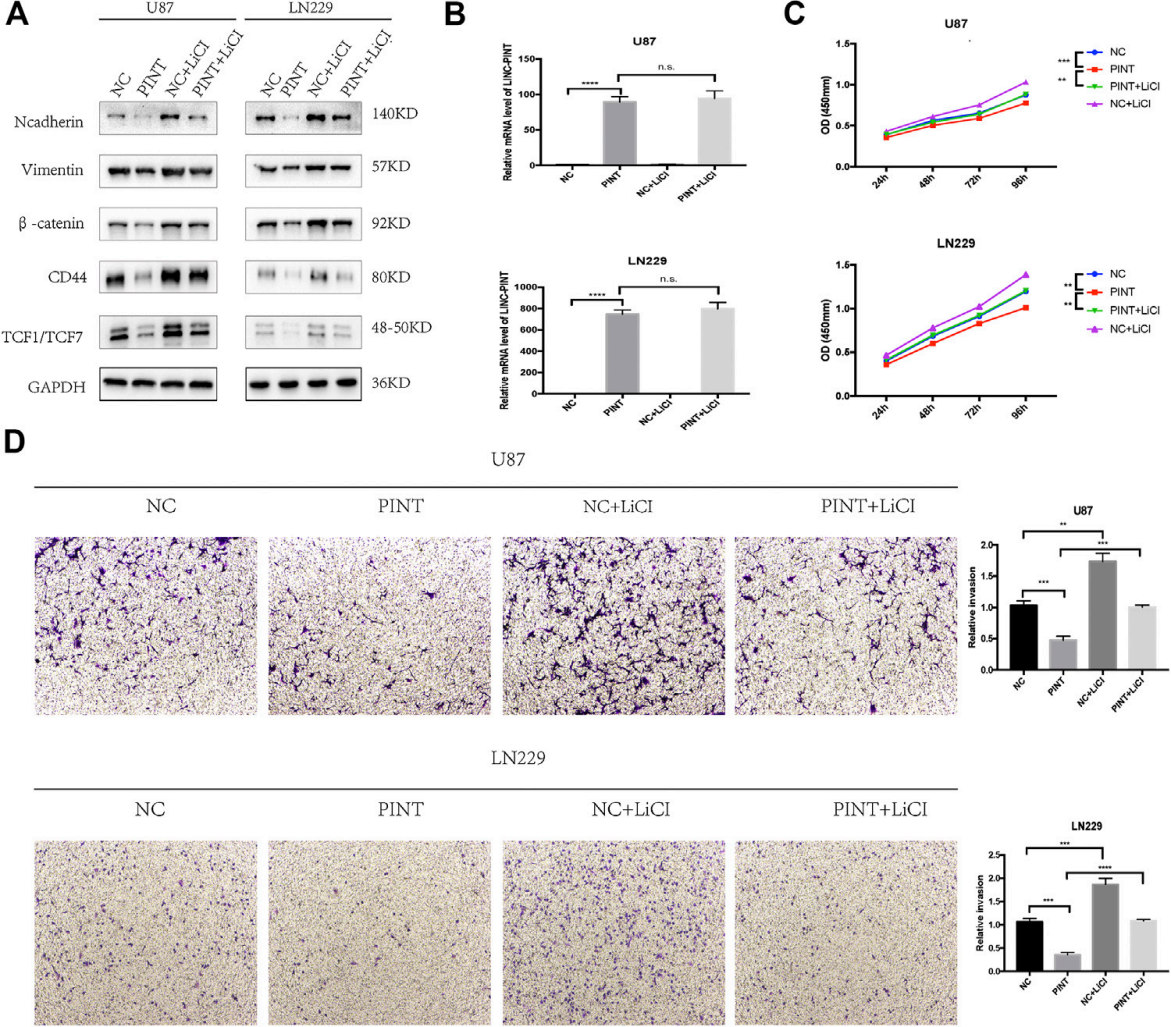
LINC-PINT suppressed EMT, tumor proliferation, and invasion by blocking Wnt/β-catenin signaling pathway in GBM. **(A)** Western blot assay for EMT markers N-cadherin and Vimentin and Wnt/β-catenin markers β-catenin, CD44, and TCF1/TCF7 detected in transfected U87 and LN229 cells with or without Wnt/β-catenin pathway activator LiCL (20 mmol/L, treated for 24 h). **(B)** Relevant expression level of LINC-PINT in transfected U87 and LN229 cells treated with or without LiCL was determined by qRT-PCR assay. **(C)** The cell viability of transfected U87 and LN229 cells treated with or without LiCL was detected by CCK8 assay. **(D)** The cell invasion ability of transfected U87 and LN229 cells treated with or without LiCL was detected by transwell assay. n.s., no significance. **p < 0.01, ***p < 0.001, ****p < 0.0001.

It has been confirmed that Wnt/β-catenin pathway has great influence on EMT process in different kinds of tumors including GBM (Zhang et al., 2012; He et al., 2019). The previous experiments verified that LINC-PINT inhibited EMT and Wnt/β-catenin signaling in GBM cell lines. To further determine whether LINC-PINT inhibited EMT through Wnt/β-catenin signaling. We used Wnt/β-catenin signaling activator LiCl to check if there was a rescue effect. It turned out that LiCL could retrieve the expression changes of the EMT mesenchymal makers N-cadherin and Vimentin induced by LINC-PINT (Figure 7A). In addition, qRT-PCR assay showed that LiCL did not affect the expression of LINC-PINT in U87 and LN229 GBM cell lines (Figure 7B), indicating that LINC-PINT was in the upstream of Wnt/β-catenin pathway. Additionally, as revealed in CCK8 (Figure 7C) and transwell experiment (Figure 7D), LiCL could recover the inhibition effect of LINC-PINT on GBM cell proliferation and invasion. Together, our study confirmed that LINC-PINT suppressed EMT as well as tumor proliferation and invasion via inhibiting Wnt/β-catenin signaling pathway in GBM.

## Discussion

GBM represents the most aggressive glioma with high invasive potential, infiltrating deeply into the surrounding brain parenchyma. Patients who suffered from GBM are reported to have a dismal prognosis and poor survival time even with the progress in current treatment including surgery, chemotherapy, and radiotherapy (Alexander and Cloughesy, 2017). Though GBM tissues attribute to the mesenchymal subtype, recent studies have shown the relevance of EMT or EMT(-like) mechanisms to increasing the malignancy and invasiveness of nonepithelial tumors including GBM (Kahlert et al., 2013; Iser et al., 2017). Targeted inhibition of EMT is of great perspective in GBM treatment. LncRNAs have been verified to play a pivotal role in human disease including GBM by an increasing number of studies. For example, LncRNA MIR4435-2HG potentiates the proliferation and invasion of glioblastoma cells via modulating miR-1224–5p/TGFBR2 axis (Xu et al., 2020). LncRNA MATN1-AS1 prevents glioblastoma cell from proliferation and invasion via RELA regulation and MAPK signaling pathway (Han et al., 2019), suggesting that LncRNAs could be used as diagnostic biomarkers and potential therapeutic targets in GBM in the future. Moreover, few LncRNAs have been reported to mediate the EMT process in GBM. For example, Jia et al. (2018) reported that silencing of LncRNA-H19 decreases chemoresistance of GBM cells to temozolomide by suppressing EMT via the Wnt/β-catenin pathway. Zhang et al. (2017) verified that LncRNA HOTTIP promotes hypoxia-induced EMT of GBM by regulating the miR101/ZEB1 axis. Zeng et al. (2017) showed that knockdown of LncRNA CCAT2 inhibits cellular proliferation, invasion, and EMT in GBM. However, the potential biological function and molecular mechanisms of lncRNA-mediated EMT in GBM remain largely unknown.

Through bioinformatic analysis, we noticed a LncRNA LINC-PINT, which was downregulated in GBM tissues and associated with good prognosis in GBM patients. Further gene relevance analysis predicted that LINC-PINT had negative correlation with EMT related genes N-cadherin, Vimentin, and Slug in GBM. In previous study, LINC-PINT has been verified to act as a tumor suppressor in various kinds of cancers. Marin-Bejar et al. discovered that LINC-PINT inhibited cell invasion of colorectal and lung adenocarcinoma through a highly conserved sequence. Feng et al. (2019) reported that LINC-PINT inhibited tumor cell proliferation, invasion, and migration in gastric carcinoma by interacting with miR-21. Zhang et al. (2019) showed that LINC-PINT acted as a tumor suppressor by sponging miR-543 and miR-576–5p in esophageal cancer. Xu et al. (2019c) verified that LINC-PINT suppresses cell proliferation and migration of melanoma via recruiting EZH2. Interestingly, in clear cell renal cell carcinoma, LINC-PINT could also exert an oncogenic role. Duan et al. (2019) discovered that LINC-PINT promoted proliferation through EZH2 and predicts poor prognosis in clear cell renal cell carcinoma. But to date, the relevance and underlying mechanisms of LINC-PINT in glioblastoma have not been explored yet.

Experimentally, we performed qRT-PCR assay to test the expression level of LINC-PINT in GBM cell lines LN229, U87, A172, T98, U373, U251, and U118 and normal human astrocytes SVG p12. And it turned out that the expression of LINC-PINT was lower in GBM cell lines than in normal human astrocytes, consistent with the GEPIA database. Then we carried out gain and loss assay of LINC-PINT to further explore the biological functions of LINC-PINT in glioma. The colony formation and CCK8 assay demonstrated that LINC-PINT inhibited cell proliferation and viability in U87 and LN229 GBM cell lines. At the same time, as shown in cell invasion and wound healing assay, LINC-PINT inhibited cell invasion and migration of GBM. What’s more, tumor xenograft experiment and tumor peritoneal metastasis experiment showed that LINC-PINT could also inhibit cell proliferation and invasion in GBM *in vivo*.

**TABLE 1:** The sequences (5–3′) of silencer for LINC-PINT (si-PINT).

si-PINT1	ASO1	GGA​AAA​TGG​AAG​AAA​CGG​AG
	siRNA1	AGC​AGA​ATA​AAC​CAC​TGA​A
si-PINT2	ASO2	CAG​AGT​ATG​TAC​TTC​TCA​AC
	siRNA2	GAA​CCA​TCT​CAG​AAG​CCA​T
si-PINT3	ASO3	GTG​GAT​GCT​TTG​TTT​CTC​AA
	siRNA3	GGA​ACT​TGC​CCT​GTC​TCC​T

The GO analysis indicated that one of the vital biological functions LINC-PINT has was to activate the cell adhesion molecule binding. As widely recognized, losing cell adhesion contact is the main feature of epithelial-mesenchymal transition, which implied the potential connection between LINC-PINT and EMT. What’s more, the gene correlation prediction from GEPIA database revealed negative correlation of LINC-PINT with EMT related markers N-cadherin, Vimentin, and Slug. So we performed western blot and immunofluorescence assay to testify the relevance of LINC-PINT and EMT in GBM cell lines. And it turned out that LINC-PINT inhibited epithelial-mesenchymal transition in GBM.

Abnormal activation of Wnt/β-catenin signaling has been reported in various tumors to promote tumor cell proliferation, migration, and invasion. Moreover, It has been confirmed that Wnt/β-catenin pathway also has great influence on EMT process in different kinds of tumors including GBM (Jia et al., 2018; Zhang et al., 2018; Liu et al., 2019). In the present study, gene correlation analysis from GEPIA website predicted that LINC-PINT had negative correlation with Wnt/β-catenin related genes. To elucidate the mechanism of how LINC-PINT regulates EMT in glioma, we further focused on Wnt/β-catenin signaling. Under normal conditions, the majority of β-catenin proteins attach on cell membrane with few existing in cell cytoplasm. The degradation of β-catenin is suppressed when Wnt/β-catenin signaling is abnormally activated. Then β-catenin enters into the nucleus of tumor cells and upregulates the downstream markers such as CD44, TCF1/TCF7, and C-Jun to drive tumor carcinogenesis or EMT related genes such as N-cadherin, Vimentin, and Slug to mediate EMT process (Zhan et al., 2017). The western blot and immunofluorescence assay confirmed that LINC-PINT suppressed the expression of the proteins related to Wnt/β-catenin pathway including the key effector β-catenin, the nuclear transcription factor TCF1/TCF7, and the downstream targets C-Jun and CD44, proving that LINC-PINT inhibited Wnt/β-catenin signaling in GBM. To further determine whether LINC-PINT inhibited EMT through Wnt/β-catenin signaling, we used Wnt/β-catenin signaling activator LiCl to check if there was a rescue effect. It turned out that LiCL could retrieve the expression changes of EMT markers induced by LINC-PINT. What’s more, the CCK8 and transwell experiment also revealed similar effect that LiCL could reverse the inhibition effect of LINC-PINT on GBM proliferation and invasion. In addition, qRT-PCR assay showed that LiCL did not affect the expression of LINC-PINT in U87 and LN229 GBM cell lines. Taken together, LINC-PINT was in the upstream of Wnt/β-catenin pathway and suppressed tumor proliferation, invasion, and EMT by blocking Wnt/β-catenin signaling in GBM.

In this study, we should also pay attention to the possible limitation that the direct targets of LINC-PINT in Wnt/β-catenin signaling are unknown and need further exploration. As previously reported, lncRNA NEAT1 promoted GBM tumorigenesis by serving as a scaffold to recruit EZH2 to silence the target genes Axin2, ICAT, and GSK3B to promote Wnt/β-catenin signaling (Chen et al., 2018). LncRNA PTCSC3 inhibits the proliferation and migration of GBM through suppressing Wnt/β-catenin signaling by targeting LRP6 (Xia et al., 2017). Through binding to β-catenin, Linc00320 inhibits Wnt/β-catenin signaling by disrupting β-catenin binding to TCF4 in Glioma cells (Tian et al., 2019). In the absence of Wnt ligands, cytoplasmic β-catenin is recruited and degraded by a destruction complex APC/axin/GSK-3β. When Wnt ligand binds to Fz and LRP5/6, the destruction complex is disassembled; then cytoplasmic β-catenin accumulates and translocates to the nucleus, binding the transcription factor TCF/LEF to activate the downstream genes and EMT related targets (Zhan et al., 2017; Hu et al., 2018). LINC-PINT could mediate Wnt/β-catenin in any of the above steps, which is the unclear mechanism we need to further explore. What’s more, it is a limitation that there were no qRT-PCR data of GBM tissues, which is the problem we need to solve next.

## Conclusion

In conclusion, our study first demonstrated that LINC-PINT was downregulated in GBM cell lines and could suppress tumor proliferation, invasion, and epithelial-to-mesenchymal transition by blocking Wnt/β-catenin signaling in GBM. Our findings showed a novel regulation mechanism of LINC-PINT/Wnt signaling/EMT axis in GBM, providing new perspectives into the pathogenesis and invasiveness of glioblastoma and verifying LINC-PINT as a potential prognostic biomarker and novel therapeutic target in GBM.

**TABLE 2:** The sequences (5′–3′) of qRT-PCR primers.

LINC-PINT	Forward	GAA​CGA​GGC​AAG​GAG​CTA​AA
	Reverse	AGC​AAG​GCA​GAG​AAA​CTC​CA
Actin	Forward	CTC​CAT​CCT​GGC​CTC​GCT​GT
	Reverse	GCT​GTC​ACC​TTC​ACC​GTT​CC

## Data Availability Statement

The raw data supporting the conclusions of this article will be made available by the authors, without undue reservation.

## Ethics Statement

The animal study was reviewed and approved by the Research Ethics Committee of Xinhua Hospital.

## Author Contributions

ZS and YM conceived and designed the experiments. ZS and SL performed all the experiments and analyzed the data. ZS, CZ, and TC wrote and revised the manuscript. All authors read and approved the final manuscript.

## Funding

This study was supported by the grants from the National Natural Science Foundation of China (No. 81701234) and the Foundation for Interdisciplinary Research of Shanghai JiaoTong University (YG2015MS65).

## Conflict of Interest

The authors declare that the research was conducted in the absence of any commercial or financial relationships that could be construed as a potential conflict of interest.
